# A Mosquito-Proof Dwelling-House

**Published:** 1900-05-19

**Authors:** 


### A MOSQUITO-PROOF DWELLING-HOUSE.

It is arranged that Dr. Sambon and Dr. G. C. Low, with their servants, shall live in the most malarious part of the Roman Campagna during the worst season of this year—namely, from June till October—with the object of testing the possibility of keeping free from the disease by the simple method of keeping within a mosquito-proof house during the hours when mosquitos bite—namely, from an hour before sunset to an hour after sunrise. The anti-malaria or mosquito-proof hut in which they are going to sleep during this experiment has a roof which overhangs the walls to a distance of about three feet, by which means the walls and the window openings are much protected from the sun's rays. The windows are protected from the entrance of mosquitos by permanent wire gauze screens, and the entrance by having double doors with a mosquito curtain between them. Under the overhanging eaves a space is left all round the house for ventilation, and this also is protected by wire gauze. In regard to the floor and walls and roof every care has been taken not only to ensure coolness, as far as may be, but to do this without any openings being left through which mosquitos might gain access. The experiment is being tried under the auspices of the English Colonial Office, and if it can be shown that by so arranging the dwelling-house as to keep out the gnats, and by keeping within doors during the hours when these insects are in the habit of biting, people can protect themselves from malaria without otherwise interfering with their ordinary avocations during the daytime, a great step will have to be taken in the prevention of the disease. It is understood that if this experiment should turn out well the Colonial Office will arrange for similar mosquito-proof dwellings to be provided for all Colonial servants in Africa. Probably the same will be done in regard to the malarial districts in India.

